# Women’s Experiences of Physical Features in a Specially Designed Birthing Room: A Mixed-Methods Study in Sweden

**DOI:** 10.1177/19375867221077097

**Published:** 2022-03-16

**Authors:** Lisa Björnson Skogström, Emma Vithal, Helle Wijk, Göran Lindahl, Marie Berg

**Affiliations:** 1 Institute of Health and Care Sciences, Sahlgrenska Academy, University of Gothenburg, Sweden; 2 Department of Obstetrics and Gynecology, Sahlgrenska University Hospital, Gothenburg, Sweden; 3 Centre for Healthcare Architecture (CVA), Chalmers University of Technology, Gothenburg, Sweden; 4 Division of Building Design, Department of Architecture and Civil Engineering, Chalmers University of Technology, Gothenburg, Sweden; 5 Department of Quality Assurance and Patient Safety, Sahlgrenska University Hospital, Gothenburg, Sweden

**Keywords:** birthing room, childbirth experience, evidence-based design, healthcare environment, high-income country, mixed-method

## Abstract

**Aim:**

To explore women’s experiences of physical features in a birthing room designed to be adaptable to personal wishes and needs during labor and birth.

**Background:**

Childbirth is a central life event influenced by numerous factors, including the healthcare environment; however, there is insufficient knowledge on how the physical design affects women during birth.

**Methods:**

This study was part of a randomized controlled trial in the Room4Birth research project, including women randomized to receive care in a new birthing room designed with physical features changeable according to personal wishes. Data consisted of responses to two questions analyzed with descriptive statistics (*n* = 202) and semi-structured interviews analyzed for content (*n* = 19).

**Results:**

A total of 93.6% (*n* = 189) assessed the physical features in the birthing room as meaningful to a very high or high extent. The overall impression of the room was positive and exceeded women’s expectations. They felt welcomed and strengthened by the room, which shifted the focus to a more positive emotional state. The room differed from traditional hospital birthing rooms, contained familiar features that maintained integrity, and had space for companions. The variety of physical features was appreciated. Of nine listed physical features, the bathtub was ranked most important, followed by the projection of nature scenery, and dimmable lighting, but the room as a whole appeared most important.

**Conclusions:**

When planning and designing hospital-based birthing rooms, it is crucial to offer possibilities to adapt the room and physical features according to personal wishes.

Giving birth is a central life event following women through their lives (Simkin, 1991). The experience is individual and complex, influenced by social, environmental, organizational, and policy contexts (Larkin et al., 2009). It is also influenced by factors such as the woman’s age, parity, fear, self-efficacy, control, preparation, expectations, experience of pain (Hosseini Tabaghdehi et al., 2020), and degree of participating in decision-making (Gibbins & Thomson, 2001; Henriksen et al., 2017). To have a trustful relationship with an attending midwife, and to get support from a birthing partner, is also crucial for a positive childbirth experience (Karlström et al., 2015; Nilsson et al., 2013). Also influential are the occurrence of medical interventions and complications, such as induction of labor, prolonged labor, instrumental vaginal birth, emergency cesarean section, and complications related to the baby leading to a transfer to a neonatal care unit with separation of mother and baby (Hosseini Tabaghdehi et al., 2020).

The physical environment can influence labor outcome and women’s experience of labor and birth (Nielsen & Overgaard, 2020; Nilsson et al., 2020; Setola et al., 2019). A birth environment perceived by a birthing woman as private, safe, and undisturbed has been mentioned as important for the labor and birth progress to be physiologically normal, as well as reducing the occurrence of medical interventions (Buckley, 2015). Such a birth environment promotes the release of endogenous oxytocin, which has numerous beneficial effects, such as enhancing labor contractions and well-being and reducing fear, anxiety, and pain. A birth environment experienced as unfamiliar and stressful can in contrast decrease the release of endogenous oxytocin, which in turn inhibits contractions and thus necessitates medical intervention (Uvnäs-Moberg et al., 2019).

***A birth environment perceived by a birthing woman as private, safe, and undisturbed has been mentioned as important for the labor and birth progress to be physiologically normal***

The scientific evidence on effects of the physical aspects in a birthing room is yet sparse. A systematic review identified several aspects in a birthing room that positively influence maternal and neonate physical and emotional outcomes. Means of distraction, comfort, and relaxation, including images of nature displayed on a screen to support distraction, makes women’s experiences of labor and birth more pleasant. Features of familiarity have been associated with shorter length of labor and decreased intensity of labor pain. A room enabling the women to move around have been found to influence their sense of domesticity, which in turn was related to reduced duration of labor and women’s experiences of labor pain. Diminishment of medicotechnical equipment has been found to be related to decreased fear of childbirth and of experiencing childbirth as a critical event (Nilsson et al., 2020). A systematic synthesis of qualitative research on issues related to place and space of childbirth identified the necessity of creating a birthing space that is more than a welcoming physical space, and that positions the birthing woman at the center by supporting her needs, desires, and philosophy of birthing. The women’s needs in relation to space of childbirth was found to be underpinned by four aspects: a homey space, a spiritual space, a safe space, and a territorial space (Carlsson et al., 2020). These findings are in line with a review on the influence of environmental factors on patients, where environmental factors were found to influence patients’ physical and psychological recovery, feeling of satisfaction, including factors such as space, views of nature, and experience of privacy. However, no studies included women in labor and birth (Ulrich et al., 2008). A later study shows that the healthcare environments should strive to be adaptable to meet the needs of every unique patient and their companion (Nordin et al., 2017).

The influence of hospital-based birthing rooms is insufficiently studied, and there is need to achieve a greater understanding of women’s experiences related to the design of birthing rooms. As part of a research project labeled Room4Birth (R4B), the objective of the study presented in this article was to explore women’s experiences of physical features in a hospital-based birthing room designed to be more adaptable to personal wishes and needs during labor and birth.

## Method

### Design

A mixed-methods study with an explanatory, two-phase sequential design was conducted based on methodology described by Creswell and Plano Clark (2017). Phase 1 had a quantitative approach investigating women’s opinions about certain physical features through closed, directed questions. Phase 2 had a qualitative approach with semi-structured interviews with strategically chosen women answering the questionnaire to gain a deepened understanding of the women’s opinions about certain physical features.

### Setting

As a part of the Room4Birth research project, a randomized controlled trial (RCT) was conducted at one of the three labor wards at a university hospital in western Sweden. Inclusion criteria for study participants in the RCT were women in spontaneous start of labor, ≥18 years of age, and classified as “Robson 1,” that is, nulliparous at ≥37 gestational weeks, with a single live fetus in cephalic presentation and in spontaneous labor. Women with induced labor, planned cesarean section, and multiple gestation or in latent phase of labor were excluded. Women agreeing to participate in the RCT study were randomized to care either in a regular birthing room or in a refurbished birthing room, the so called new room with more physical features to increase the adaptation of the room to personal wishes and needs. A study protocol of the RCT has been published (Berg et al., 2019). The study took place between January 2019 and October 2020, with some episodes of disruptions due to consequences of the COVID-19 pandemic situation and some technical problems. The study ended earlier than planned due to effects related to COVID-19. The main findings of the RCT will be published in a separate paper. This study includes the study participants randomized to the new room.

***As a part of the Room4Birth research project, a randomized controlled trial (RCT) was conducted at one of the three labor wards at a university hospital in western Sweden***

**Figure 1.**
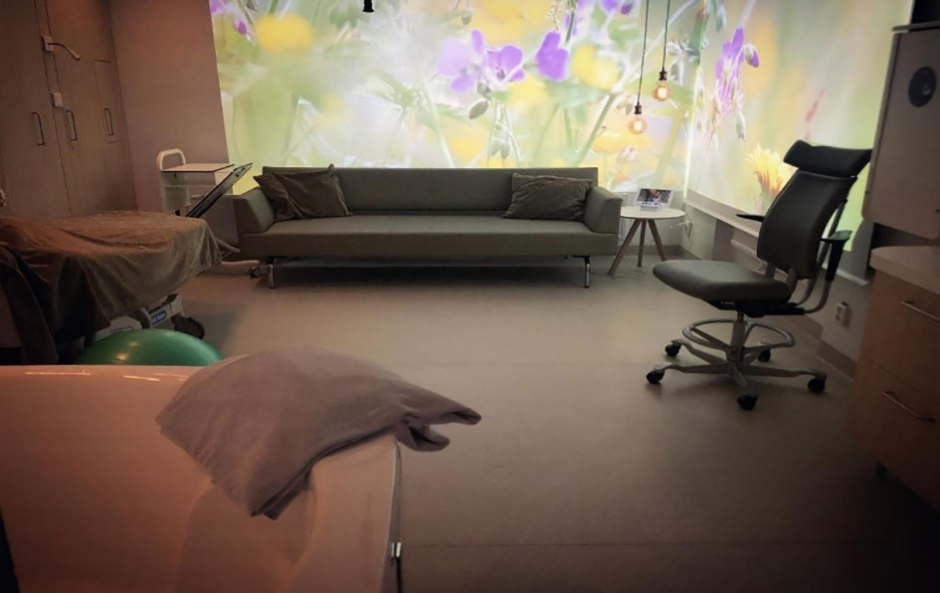
Part of the new room designed to be adaptable to birthing women's wishes and needs. *Source*: Photo by Lisa Björnson Skogström.

The new room (see Figure 1) had a size of 23.8 m^2^ (256 ft^2^) including an entrance hall with a drapery and a bathroom with a toilet and shower. It had the same medicotechnical devices as in the regular rooms, but hidden behind wooden panels that could be rolled up if needed. The illumination had several options for dimming, and a 40 mm suspended sound absorber was installed in the ceiling. There was also a window with curtains, an ordinary birthing bed positioned alongside the wall and covered with a bedspread, a sofa with soft pillows that could be converted to an extra bed, and a chair for a birthing companion, designed for comfort and with adjustable height. Furthermore, the new room had a bathtub, a birth ball, a birth support rope, and a cabinet for personal belongings. A media installation and its screen covered two walls and the window, offering seven different choices of programmed nature scenery such as forest or ocean, with light, sound effects, and music. See details of the new room, in Table 1.

**Table 1.** Details of the New Room.

Content	New Room
Size	23.8 m^2^ (256 ft^2^)
Entrance hall	Yes, 3 m^2^ (32,3 ft^2^)
Toilet with shower	Yes
Bathtub	Yes
Window, opening	Yes, hidden if media installation in use
Lighting	Yes, several options with dimming feature
Sound absorber	Yes, a 40 mm (0.13 ft) suspended sound absorber in the ceiling
Media installation	Yes, installation covers two walls, including the window, and offers choice of programmed nature scenes with light, sound effects, and music
Birthing bed, ordinary	Yes, covered with bedspread
Medicotechnical equipment	Yes, hidden behind a wood-panel wall, which is rolled up when necessary
Rounded corners on furniture	Yes, some
Sofa	Yes, can be converted to an extra bed for birthing companion
Chair for companion	Yes, designed for comfort, adjustable height
Mirror	Yes
Birth ball	Yes
Birth support rope	Yes
Cabinet for personal belongings	Yes, with ability to recharge electronic devices such as mobile phone

### Participants and Data Collection

#### Phase 1

After giving birth in the new room, women completed a questionnaire on a touchscreen tablet, before leaving the labor ward. The questionnaire was developed by researchers within the R4B project and contained several closed, predefined questions, of which two questions were specifically directed to women giving birth in the new room. The first question concerned whether the physical features in the birthing room were meaningful for giving birth, with four options: (i) To a very high degree, (ii) To a high degree, (iii) To a low degree, or (iv) Not at all. In the second question, the women were asked to rank nine defined physical features in the birthing room from 1 to 9 regarding their importance in relation to the childbirth, where 1 was ranked as *the most important*, and 9 as *the least important*. The feature ranked as most important (Rank 1) was assigned 9 points, and the feature ranked as least important (Rank 9) rated 1 point. In addition, the questionnaire contained questions about the women’s sociodemographic data.

#### Phase 2

In Phase 2, a purposive sampling was chosen of participants answering the questionnaire to ensure a heterogeneous sample and a diversity of opinions and experiences. The strategic sample was considered to include women who had answered differently regarding the physical features. Other aspects considered to assure variability were age, education level, cohabitation status, and country of origin, also labor outcomes such as vaginal birth, vacuum extraction, emergency cesarean section, and severe bleeding. Invited women had to speak either Swedish or English. Based on these criteria, a total of 30 women were invited via e-mail with information about the objective of participation in the study. Reminders were sent by e-mail and text messages. 19 women agreed to participate, seven declined without explanation, and four women did not reply.

An interview guide with semi-structured questions was developed by the research group, starting with an open question regarding the women’s first impression when entering the new room and a question exploring whether the women felt that they had the opportunity to adapt the room according to their personal wishes and needs. Subsequent questions were based on the women’s individual rating of the physical features in the Phase 1 questions. Interviews were performed individually by L.B.S. or E.V. via telephone and occurred one to two years after birth. The mean time of the interviews was 17 min, and they were recorded and transcribed verbatim.

The underlying research materials, related to this study, is collected and saved in a secure place and de-identified with an associated code key at the University of Gothenburg, only accessible to the principal investigator for the R4B project, Professor Marie Berg.

### Data Analysis

The two questions were analyzed with descriptive statistics, presented in percentage and means. The interviews were analyzed using content analysis with an inductive approach according to Elo and Kyngäs (2008). The content analysis was conducted by L.B.S. and E.V. with continuous feedback from the other researchers. First, the transcripts were read through several times separately, while the researchers made notes and headings in the text. Next, meaning units regarding women’s experience of the physical features were identified and inserted into a coding sheet where similar data were identified and grouped together in subcategories. The subcategories that belonged together were then grouped into two main categories describing the content. All researchers agreed on the final identification and description of the main categories and subcategories.

### Preunderstanding

We all together in the research group have preunderstandings that may have influenced this research. We have, however, systematically challenged our respective preunderstandings through continuous critical discussion during the whole research process. L.B.S. and E.V. are registered midwives, working R4B project, have both worked at the labor ward where the RCT was conducted, and had taken care of study participants randomized to both types of rooms, the new room and regular rooms. M.B. is a senior consultant registered midwife, is principal investigator for the R4B project, and has conducted numerous studies around labor and birth. H.W. is a senior consultant registered nurse, a researcher with long experience of healthcare environmental studies and a researcher within the R4B project. G.L. is an architect focusing on planning and design of healthcare environments and a researcher in the R4B project.

### Ethical Consideration

The study was performed in accordance with ethical principles for medical research involving human subjects (World Medical Association, 1964) and was ethically approved by the regional ethical board (No. 478–18). The study participants were offered and cared for according to the same standard as other patients in the labor ward. They were informed about their right to discontinue their participation without further explanation and were also given contact information for the responsible researchers. All data were handled with confidentiality. During the execution of this study, no risks were identified, and the participants were not exposed to any treatments that were not defensible.

## Findings

Of 204 participating women randomized to the new room, 202 (99%) answered the questionnaire. Their age varied between 18 and 40 years with a mean of 29.6 years at time for giving birth (*SD* = 4.5). The 19 women interviewed were between 20 and 40 years old at time for giving birth, with a median age of 31 years. Of these interviewed women, 16 had a vaginal birth and three an emergency cesarean section. More characteristics of the participants are presented in Table 2.

**Table 2.** Participant Characteristics.

Characteristics	Phase 1 (Questionnaire) *n* (%)	Phase 2 (Interview) *n* (%)
Education	202 (100)	19 (100)
Elementary school	5 (2.47)	1 (5.26)
Upper secondary school	47 (23.27)	4 (21.05)
Postsecondary education	150 (74.26)	14 (73.68)
Cohabitation status	202 (100)	19 (100)
Living with partner	191 (94.55)	15 (78.94)
Living alone	5 (2.48)	3 (15.79)
Other	6 (2.97)	1 (5.26)
Area of origin	202 (100)	19 (100)
Sweden	149 (73.76)	14 (73.68)
Other country in Europe	29 (14.35)	3 (15.79)
Asia	16 (7.92)	1 (5.26)
Africa	7 (3.47)	1 (5.26)
South America	1 (0.50)	0
Years of living in Sweden, if originating outside Sweden	53 (100)	5 (100)
<10 years	19 (35.85)	3 (60)
≥10 years	34 (64.15)	2 (40)

Concerning the meaningfulness of the physical features in the birthing room, 64.9% (*n* = 131) assessed it as meaningful to a very high extent, 28.7% (*n* = 58) to a high extent, 5.4% (*n =* 11) as low, and 0.99% (*n =* 2) as not at all. The ranking of the nine physical features in the birthing room identified the bathtub as most important (6.7 points), followed by the projection of nature scenery on the walls with a combination of light, sound effects, and music (6.3 points) and the dimmable lighting (6.01 points). Mean ranking of nine features in the new room are presented in Table 3.

**Table 3.** Mean Ranking of Nine Features in the New Room.

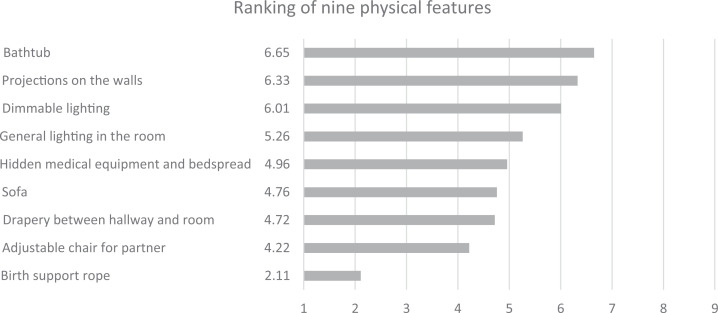

The 19 women’s experiences of the birthing room are described in three main categories, two of them with subcategories (Table 4).

**Table 4.** Main categories and subcategories describing women’s experiences of the birthing room.

Main Categories	Subcategories
A positive impression	Welcoming and strengthening Offering a familiar environment
Opportunities offered by the room	A variety of features promoting a positive emotional state A maintained integrity A place for the birthing companion
Limitations and areas of improvement	

### A Positive Impression

The overall impression of the room was more important than specific physical features, since the different physical features reinforced one another. All women, despite different needs, were grateful for the possibility to give birth in the new room. This concerned both those who had wished to give birth at home or even outdoors in the nature, and those with fear of childbirth.

I think it was the entire room. When first entering the room, I felt this is the room of opportunities, providing the best birthing experience possible. To adjust it according to one’s needs. (Participant 12)

I still think that I was very lucky to get that room. Before giving birth I was very scared of how it would be? But now, whenever I think about my birth, I feel happy about it. I feel that it was one of the best experiences that I ever had, that will stay with me forever. The birth of my first child was a very, very nice experience. (Participant 15)

### Welcoming and Strengthening

The room gave a feeling of being welcomed and feelings of happiness and gratitude. Further adjectival expressions of the room were magic, wonderful, fantastic, clean, and luxurious. The room provided a sense of calmness and was experienced as nice and comforting, which contributed to being able to relax and focus on letting the body do the work.

It [the room] felt inviting and pleasant, like it was made for me to be able to relax. (Participant 7)

Furthermore, the room strengthened the women. They felt in control and as though they owned the room; it was “their place” during birth. The room as a whole provided the women with several options, and they felt free to change it according to their own needs in order to have a good birthing experience. The strengthening aspect of the room was described by one participant:

I think it [the room] made me a lot stronger during birth. It maybe sounds strange, but you are dependent on the surrounding environment. It influences you and the feeling of strength and safety. (Participant 6)

### Offering a Familiar Environment

The new birthing room offered a familiar, homey, and private environment that made the women feel safe and calm. The room was described as like a hotel room, a spa, or an oasis. There were several features that made the birthing room feel familiar. This included the homey decor, the furnishings, and textiles. It also included the dimmable lighting in contrast to the bright lighting in other parts of the labor ward, and the sound absorber that buffered noise from the outside.

When the women arrived at the labor ward, they first entered an examination room with a traditional hospital design where a midwife assessed whether the women were in active labor. If so, they were transferred to the new room, which was much bigger and more spacious. The women expected to enter a traditional hospital birthing room design, described as, a sterile environment with bare white walls, bright lighting, and visible medical equipment. They were therefore pleasantly surprised by the nontraditional design in the new room:

I personally was scared that the sterile hospital environment would make me stressed, obstruct the flow of oxytocin and thus inhibit the process…. So, I was very happy that I got this environment. (Participant 7)

The wooden panels hiding the medical equipment were highly appreciated, as it gave a calmer impression of the room through eliminating signals of danger, worst-case scenario, and sense of illness. It diminished stress and evoked feelings of the possibility for a pleasant labor.

For me, the features in this room aimed to counteract what I was afraid of would stress me in an ordinary hospital environment…. So to diminish this sterile, white and bare room with visible medical instruments…. For me it [the new room] was just great! I felt so much calmer and more welcomed in such a physical environment. It felt like the focus was on the correct thing, meaning that the focus was on helping me, as a mother, that my body could do its thing instead of focusing on everything that could go wrong…which I think would have hampered me. (Participant 7)

Nevertheless, knowing that medical equipment was available in the room, to be used if needed, was expressed as important for some women. To decrease feelings of stress and fear, they needed to be assured that all technology was available. One woman, with fear of childbirth, had an agreement with her obstetrician that if her fear became too severe during some stage of the labor she would have a cesarean section. She repeatedly stressed that the familiar environment probably contributed to feeling safe enough to give birth vaginally, which she did. This experience also influenced her desire to, in the event of a future pregnancy, strive for that birth to be vaginal as well.

***The wooden panels hiding the medical equipment were highly appreciated, as it gave a calmer impression of the room through eliminating signals of danger, worst-case scenario, and sense of illness.***

### Opportunities Offered by the Room

The new birthing room offered numerous opportunities. That the new room had a variety of physical features to be used according to women’s own wishes was much appreciated. The design of the room positively changed the women’s mood, maintained their integrity, and offered a place for the birthing companions.

### A Variety of Features Promoting a Positive Emotional State

The variety of physical features in the birthing room was much appreciated, and when used according to personal choices, it was a tool promoting a positive emotional state. All nine physical features that the women were asked to rank were mentioned in positive terms. The opportunity to use the bathtub was appreciated, even though not all the women used it. That the bathtub was placed in the birthing room was highly valued, as it enabled women to use it without leaving the room. The bathtub was perceived as user friendly, comfortable, pain relieving, calming, cozy, and with clean water because of the quick water refill. It was easy to get in and out of it. A few found it too deep. Another highly ranked physical feature was the dimmable lighting which made it possible to create a cozy atmosphere. The birth support rope was ranked as number nine, that is, least important, but women were still pleased that there was a rope if wanted.

The projection of nature scenery on the walls with a combination of light, sound effects, and music was also highly ranked, and it was easy to operate for both women and birth companions. The scenes evoked positive personal memories and were also experienced as entering another world, which in turn reduced feelings of stress. The varied nature scenery yielded different types of energy and was also experienced differently. Therefore, it was important to choose scenes according to one’s own preferences. For example, a forest could be perceived as calming, and the waves of the ocean could be destressing for some as an illustration of the contractions coming and going, while others might perceive them as stressful.

The projections helped me to find inner peace…and relaxation…and they distracted from the pain. To experience pain, and focus more on looking at these nature scenes and hearing these sounds, helped a lot in coping with the pain. (Participant 12)

Oh how nice it [the nature projection] was!…The focus changed me from being afraid to being part of something strong. It was not just that we gave birth…but that the environment was such a big part of our experience. (Participant 6)

### Maintained Integrity

Labor and birth is a private life event in which integrity should be maintained. This was supported by the new birthing room. Several women mentioned the anxiety they had felt about the forthcoming labor and birth, and about being naked and exposed to other people, not only staff but also other patients, which could happen when the door was opened toward the corridor. The drapery placed between the hallway and the birthing room supported the feeling of privacy. The strategic location of the bathtub in a corner also reduced the risk of being seen if the door opened.

The fact that it was not a door directly from the corridor and into the room, but a curtain in between, protected me from being exposed. It felt very comfortable when the staff came in and asked from behind the curtain: Can we come in? (Participant 6)

### A Place for the Birthing Companion

One important aspect of the room was that it provided a place for the companion. When the partner was comfortable, the woman could relax and focus on herself and on giving birth. There was a special chair for the companion, which could be easily moved close to the woman. There was also a sofa that could be changed to a bed, which could be used both by the woman and the companion to socialize and rest.

Throughout my labor and birth my partner was by my side. The whole birth process was made easy because of him. His comfort was very important to me because I wanted him to be with me and help me, throughout. So, I was very relaxed that he was comfortable in the room. And there was a place for him to rest as well…. Because, if he was tired, he would not be in a good mood and not be able to help me. But he had a good chance to rest and sit comfortably next to me. (Participant 15)

### Limitations and Areas for Improvement

Despite the overall positive experiences of the new birthing room, there were some limitations and areas for improvement. The most requested physical feature in the birthing room was to have a window, to have access to fresh air and daylight, and to see nature outside the room. The birthing room did in fact have a window, but when using the nature projection on the walls, the wall including a window was covered by the projection screen. However, even when not using the projection screens, the environment outside the window was only a brick wall of a building at a short distance, and no view of nature was offered. Due to the absence of a visible window and lack of daylight, feelings of being disorientated in time emerged; it was difficult to determine whether it was day or night. Other areas of improvements mentioned were to have the opportunity to play one’s own chosen music when seeing the nature scenes projected on the wall and to have more textiles such as large pillows. A few women experienced the lighting panel as too technically advanced to use by themselves, they needed help from staff to use it. Better written instructions could avoid this problem. Another feature that should be better explained is how to use the birth support rope.

## Discussion

This mixed-methods study enabled exploration of women’s experiences of features in a specially designed birthing room. Prominent findings were that almost all participating women perceived the physical features in the birthing room as meaningful in relation to their labor and birth, and that the room as a whole was experienced as very positive. These findings are similar to findings from a hospital-based RCT in Denmark, where one group of women were randomized to a specially designed birthing room with several design characteristics similar to those of the new birthing room in our study (Nielsen & Overgaard, 2020).

The new birthing room in our study gave overall a positive impression. It offered a familiar environment that was welcoming and strengthening. It offered a variety of physical features that promoted a positive emotional state, maintained integrity, and offered a place not only for the women giving birth but also for their companions.

Strengthening women’s trust in their own capacity during labor has been found to be crucial (Olza et al., 2020). The importance of the birthing companion having a space has also been found in earlier research (Nilsson et al., 2020). Another study in the R4B project also adds the importance of a familiar and comfortable birthing room design, as it symbolizes tenderness and care (Goldkuhl et al., 2021). A familiar childbirth environment is found to be nonthreatening, promotes relaxation, and enhances women’s confidence (Carlsson et al., 2020). One aspect that reduced a threatening feeling in the new birthing room studied was having the medical equipment hidden behind wooden panels, which diminished stress and contributed to a familiar environment, in turn evoking feelings of safeness and calmness.

The new birthing room offered numerous opportunities for the women to use the features in the room according to their wishes and needs. The women appreciated the availability and variety of physical features, both for feeling relaxed and to decrease pain. Of nine physical features that the participants in our study were asked to rank in terms of importance, the bathtub was ranked most important, followed by the projection of nature scenery on the walls, and dimmable lighting. Feeling relaxed during labor is beneficial for the activation of the parasympathetic nervous system, which contributes to a release of oxytocin (Buckley, 2015). Being able to operate the features independently of the staff was also appreciated. These findings in our study are in line with a review showing that access to a bathtub during birth is perceived as helpful for pain relief. Further, letting the woman take control over the level of light in the birthing room is an easy way to provide women with control over the environment (Jenkinson et al, 2014).

The variety of features of the new birthing room in our study was appreciated, but a few features could be improved, the most prominent to change is to have a visible window. The women did not see the window, when it was hidden behind the projection screens showing nature scenes, which almost all women used all the time. It is evident that a view of nature and daylight can enhance well-being (CVA, 2021). It is also important that the size and placement of the window must protect patients’ privacy (Jenkinson et al., 2014). When designing birthing rooms, it should be taken into account that the window should not be covered by a projecting screen and that women should be able to see nature outside a window and not only another building. It is also important, where projections can cover two walls, to inform the women and their companions about the possibility of using projections on only one of the walls and to have clear instructions on how to turn off the one covering the window and only use the one covering a plain wall. Another desire expressed by the women was to use their own chosen music instead of the programmed music that was part of the nature screening on the walls. The negative effects of noise in hospital environments on health outcomes are widely understood, in opposite the therapeutic effects of music can be profound and research in this area is growing. Music therapy can decrease sensation of pain and anxiety in women giving birth (Jenkinson et al., 2014). This would be easy to solve in birthing room and thus to write instructions about, so that the couple could easily exercise these choices.

### Strengths and Limitations

First, we need to stress that although the study provided a lot of understanding about how women experience the physical features in a birthing room, the physical environment is not the only factor that affects women during labor and birth. Earlier research in the R4B project showed that the birthing room design can either contribute to or prevent midwives from supporting a healthy and normal physiological birth. The midwife must be present, make the room private and homey, and support the mother to be active and protect her from disturbing elements both inside and outside the room (Andrén et al., 2021). Yet, another R4B study identified that the birth environment consists of the physical birthing room, the human encounter within it, and the institutional context where women give birth. It seems like the midwives could have a strong impact on women’s activities and experiences of the birth environment (Goldkuhl et al., 2021).

One challenge with this study is that the two questions in the questionnaire were not validated, which made them less reliable, although it is a strength that the questions were developed by the experienced R4B research group. The list of nine defined physical features did not cover all features in the room. Another weakness, which also was mentioned by the participants, is that they were not able to rank the features, as they altogether gave a synergy effect and thus were not always possible to rank one by one.

A strength of the study, which partly compensates for the weakness of not having validated questions, is its mixed-method design, as this allowed for added understanding by posing more open questions in the semi-structured individual interviews, giving a rich insight into women’s experiences. That the participants were interviewed between one and two years after giving birth was not assessed as problematic. Rather, we obtained information about what had remained in the women’s memory a long time after being in the birthing room. The women did not have any difficulties remembering details or experiences from the birth. This is in line with previous findings, indicating that women can have a detailed and vivid memory of their own labor for several years after giving birth (Simkin, 1991).

## Conclusion

The study found that a hospital-based birthing room design, with physical features adaptable to personal wishes and needs, was beneficial and supportive for women during their labor and birth. The birthing room contributed to a positive impression, as it made women feel welcomed and strengthened. The expectations of the room were exceeded, as it differed from a traditional hospital birthing room and contained familiar features. The new room provided interaction with the environment according to one’s wishes, supported a shift of focus from pain toward a more positive emotional state as well as maintaining integrity, and offered a place for the birthing companion. There were a few areas for improvement, not least the need of having a visible window through which nature could be seen. The overall synergic effect of the physical features was more important than single physical features.

***The expectations of the room were exceeded, as it differed from a traditional hospital birthing room and contained familiar features.***

Altogether, the study results provide more knowledge about the role of the physical environment in a birthing room, which should be taken into account when designing new labor wards or refurbishing existing ones. As this study was conducted only in Sweden, it is important to study the needs and experiences in other contexts and to strengthen the basis for evidence-based design. Finally, we need to stress that although the study provided a lot of understanding about how women experience the physical features in a birthing room, the physical environment is not the only factor that affects women’s experience of labor and birth, but it is an important piece of the puzzle in the mapping of what influences women’s experience of childbirth.

## Implications for Practice

- Giving birth is a private life event in which integrity should be maintained. This can be supported by designing birthing rooms with strategically placed features, for example, a bathtub behind a corner as well as a drapery placed between the hallway and the birthing room since it can maintain the feeling of privacy.
- Birthing rooms, which provide interaction with the environment according to women’s wishes and needs, can support a shift of focus from pain and fear toward a more positive emotional state.
- A birthing room designed with medical equipment hidden behind wooden panels can diminish stress and contribute to a familiar environment, which in turn can evoke feelings of safeness and calmness in birthing women.
- When designing birthing rooms, it should be taken into account that windows should not be covered by projecting screens, instead the windows should enable a view of nature outside, which can enhance well-being.
- The overall impression of the environment seems to be more important than individual physical features in birthing rooms, since the different physical features can reinforce one another and achieve a synergetic effect improving the women’s experience of giving birth.
